# Cancer Metastases to the Liver: Mechanisms of Tumor Cell Colonization

**DOI:** 10.3390/ph17091251

**Published:** 2024-09-23

**Authors:** Wiktoria Andryszkiewicz, Piotr Misiąg, Anna Karwowska, Zofia Resler, Aleksandra Wojno, Julita Kulbacka, Anna Szewczyk, Nina Rembiałkowska

**Affiliations:** 1 The Students’ Research Group, No. 148., Faculty of Medicine, Wroclaw Medical University, Pasteura 1, 50-367 Wroclaw, Poland; wiktoria.andryszkiewicz@student.umw.edu.pl (W.A.); piotr.misiag@student.umw.edu.pl (P.M.); anna.karwowska@student.umw.edu.pl (A.K.); zofia.resler@student.umw.edu.pl (Z.R.); aleksandra.wojno@student.umw.edu.pl (A.W.); 2 Department of Molecular and Cellular Biology, Faculty of Pharmacy, Wroclaw Medical University, Borowska 211A, 50-556 Wroclaw, Poland; julita.kulbacka@umw.edu.pl

**Keywords:** liver metastasis, hepatic niche, tumor microenvironment, liver sinusoidal endothelial cells, Kupffer cells, matrix metalloproteinases

## Abstract

The liver is one of the most common sites for metastasis, which involves the spread from primary tumors to surrounding organs and tissues in the human body. There are a few steps in cancer expansion: invasion, inflammatory processes allowing the hepatic niche to be created, adhesions to ECM, neovascularization, and secretion of enzymes. The spread of tumor cells depends on the microenvironment created by the contribution of many biomolecules, including proteolytic enzymes, cytokines, growth factors, and cell adhesion molecules that enable tumor cells to interact with the microenvironment. Moreover, the microenvironment plays a significant role in tumor growth and expansion. The secreted enzymes help cancer cells facilitate newly formed hepatic niches and promote migration and invasion. Our study discusses pharmacological methods used to prevent liver metastasis by targeting the tumor microenvironment and cancer cell colonization in the liver. We examine randomized studies focusing on median survival duration and median overall survival in patients administered placebo compared with those treated with bevacizumab, ramucirumab, regorafenib, and ziv-aflibercept in addition to current chemotherapy. We also include research on mice and their responses to these medications, which may suppress metastasis progression. Finally, we discuss the significance of non-pharmacological methods, including surgical procedures, radiotherapy, cryotherapy, radiofrequency ablation (RFA), and transarterial embolization (TAE). In conclusion, the given methods can successfully prevent metastases to the liver and prolong the median survival duration and median overall survival in patients suffering from cancer.

## 1. Introduction

Metastasis can be defined as the spread of cancer cells to distant organs from their original site, representing the most advanced and lethal stage of cancer. The majority of cancer patients succumb to metastatic disease rather than primary tumors. Metastasis involves a series of biological events where cells from the primary tumor gradually acquire the ability to invade deeper tissues through the mucosa. Then, they disseminate through the blood and lymphatic system or by directly infiltrating neighboring structures, seed distant organs, and eventually resume proliferation at distant sites to colonize these organs [1]. The liver is highly susceptible to metastasis and is frequently affected by various common cancers. It is the second most common site for metastasis after lymph nodes, especially for gastrointestinal cancers, breast and prostate carcinomas, uveal melanoma, neuroendocrine tumors, and sarcomas. Liver metastases are also far more common than primary liver tumors. However, liver involvement in metastasis is often overlooked and under-investigated because the lesions are typically asymptomatic. Even extensive infiltration by metastatic tumors may not impact liver function or homeostasis until the disease is in its very late stages [2]. The formation of metastatic lesions depends on completing several steps, collectively known as the invasion-metastasis cascade. This cascade includes dissemination, intravasation, extravasation, transendothelial migration (TEM), and eventually colonization of the secondary organ. Although this process is largely inefficient, the primary tumor supports colonization through a mechanism called premetastatic niche (PMN) formation [3]. Our review focuses on the formation of hepatic PMN, as it is a significant site of colonization of tumor cells in the liver. Therefore, we highlight the most important data about processes leading to cancer cell colonization, including forming new vesicles supported by cytokines and growth factors and natural immune responses that are dysfunctional because of tumor cells, creating the proinflammatory processes essential for PNM to be formed [4]. We talk about metalloproteinases as they are crucial for both neovascularization and cytoskeletal rearrangements contributing to cancer cell invasion; we also focus on integrins, selectins, and Kupfer cells (KCs) that can be found in liver sinusoidal endothelial cells (LSECs) and hepatocytes as they also influence the metastasis [5,6].

## 2. Neovascularization

Tumors cannot enlarge without their blood supply, so neovascularization is essential for them to expand. The cancerous cells stimulate angiogenesis, which has a permanent role in metastases, because, firstly, it allows them to be transported to blood and then to other organs from a primary tumor and, secondly, it provides them with oxygen and nutrients [7]. The mechanism of new formation includes the excessive release of angiogenic factors such as vascular endothelial growth factor (VEGF) and intercellular adhesion molecule 1 (ICAM-1), E-selectin, and vascular cell adhesion molecule 1 (VCAM-1) regulated by VEGF [8,9]. In addition, the study showed that newly formed microvesicles will continue to form as long as VEGF is produced by tumor cells. This process can be compared to healing wounds. VEGF secretion promotes cell migration by increasing vascular permeability, supporting tumor growth by creating a temporary stroma consisting of fibrin gel, and increasing the synthesis of collagen migration of plasma proteins [10].

The liver has specific capillaries that are called LSECs. These cells play a significant role in liver homeostasis by interacting with pathogens, macromolecules, and toxins that cause pathogenesis [11]. LSECs behave like a filter between hepatocytes and blood in sinusoids. LSECs are fenestrated endothelial cells, which means they have pores that can regulate the transfer of several substances between the extravascular compartment and blood [12]. Their role can be pro- and antimetastatic. The fenestrated LCESs cannot switch their phenotype under pathological conditions and lose their fenestration, which leads to intracellular gaps. These gaps support metastasis because they facilitate the penetration of cancer cells into the liver parenchyma [13]. LCECs also become a place of fusion of platelets [14]. There are many ways in which the tumor can activate the platelets. Firstly, it releases the main agonist of platelet aggregation—thrombin. Another secreted factor is adenosine diphosphate (ADP), which promotes aggregation. These processes result in thrombosis, which protects tumor cells and leads to platelet secretion of proangiogenic factors [15]. Moreover, the platelets promote metastasis by allowing the tumor cells to escape from the central peripheral immune cells, such as natural killer cells (NK-cells), T cells, and dendritic cells (DCs). The immune escape of tumor cells is a complex process because platelets increase the ability to avoid apoptosis in many ways. Firstly, the platelets allow circulating tumor cells (CTCs) to aggregate, protecting them from NK cells and releasing interferon-γ [16]. Secondly, the study reported that transforming growth factor β (TGF-β) secreted by the platelets interacts with activating immunoreceptor natural killer group 2, member D (NKG2D) on NK cells, resulting in their decreased antitumor reactivity as the secretion of TGF-β downregulates the expression of these immunoreceptors. Moreover, the researchers of this study, by using flow cytometry and epithelial tumor cells (PC3, NCCIT, and HCT116) with platelet-rich plasma under shear stress, proved that the platelets immediately adhere to tumor cells and can be activated [17]. It is worth adding that TGF-β downregulates T-cell recruitment bispecific antibody (bsAb), leading to reduced CD4+ and CD8 + T-cell reactivity and tumor cell lysis, as these impaired cells cannot secrete perforin and degranulate properly. This leads to the conclusion that antiplatelet agents improve T-cell cancer therapy [18,19]. It is worth mentioning that tumor-induced platelet aggregation results in the secretion of other proangiogenic and protumorigenic factors. For example, secreted stromal-derived factor 1 (SDF-1) supports the facilitation of hematopoietic progenitor cells that are essential in neovascularization, and the ADP stimulates the release of VEGF [20].

This study showed that the blood vessels in metastatic liver are presented with many factors, including von Willebrand factor (vWF), α-smooth muscle actin (α-SMA), type IV collagen, laminin with the prevalence of vascular endothelial growth factor, essential fibroblast growth factor (bFGF), and acidic fibroblast growth factor (aFGF). These factors were also present in sinusoidal endothelial cells, which leads to the conclusion that they contribute to the vascularization and hyperproduction of endothelial cells. Still, the new data might confirm this statement in the future [21].

The tumor’s microenvironment consists of tumor cells and the stroma where the cells are located. The stromal cells include extracellular matrix (ECM), fibroblasts, immune and inflammatory cells, and endothelial cells, which support tumor development, including angiogenesis [22]. The stromal fibroblasts are called cancer-associated fibroblasts (CAFs). CAFs secrete cytokines, including VEGF, under the influence of interleukin-6 (IL-6) and SDF-1 [23,24]. The expression of SDF-1 promotes the recruitment of endothelial progenitor cells (EPCs) into tumor masses, leading to tumor growth [25]. CAFs are also responsible for the expression of smooth muscle actin α-SMA, a marker of myofibroblasts. Myofibroblasts are crucial for tumor angiogenesis because they are a secondary source of VEGF, significantly when tumor cells decrease their VEGF expression [26]. Moreover, CAFs contribute to tumor proliferation by secreting matrix-metalloproteinases (MMPs), including MMP-2, MMP-3, MMP-9, and MMP-13. By expressing the MMPs, type IV collagen and laminin can be degraded, which allows cancer cells to leave their primary location and facilitate in another. In addition, MMP-13 can release VEGF, which promotes angiogenesis [22,27].

## 3. The Recruitment to the Liver

It has been proven that metastasis to the liver is determined by a suitable hepatic niche, which is created by inflammatory processes [4]. Steatosis and fibrosis of the liver are factors in creating a proinflammatory state [28]. Exosomes from colorectal cancer cells carry biomolecules, which create a PMN by proliferation, inflammation, invasion, and migration, contributing to the metastasis of colorectal cancer [29].

Expressed on top of the hepatocytes, claudin-2 promotes the adhesion between hepatocytes and CTCs. Stromal cells within the primary tumor secrete IL-6 into the blood, reaching the liver. IL-6 activates signal transducer and activator of transcription 3 (STAT3) signaling in hepatocytes. Hepatocytes then release serum amyloid A1 and A2 (SAA). SAA takes part in the fibronectin and collagen buildup and the expression of proteins and chemokine ligand 6 (CCL6). SAA then causes the deposition of F4/80+ and Ly6G+ myeloid cells and liver fibrosis, creating a PMN in the liver. SAA also stimulates hepatic stellate cells (HSCs) [4].

The liver’s fibrosis and other inflammatory changes generate an infiltration of many cytokines, including chemokine ligand 21 (CCL 21), thus expanding the flow of immune cells to lymphatic endothelial cells (LECs) in the liver. Cancer cells expressing C-C chemokine receptor type 7 (CCR7) direct their movement toward CCL21 [30,31]. The chemokine gradient encourages the metastasis of tumors positive for cytokines, such as gliomas, to the liver. LECs create an immunosuppressive environment supporting tumor growth [30].

**Figure 1:**
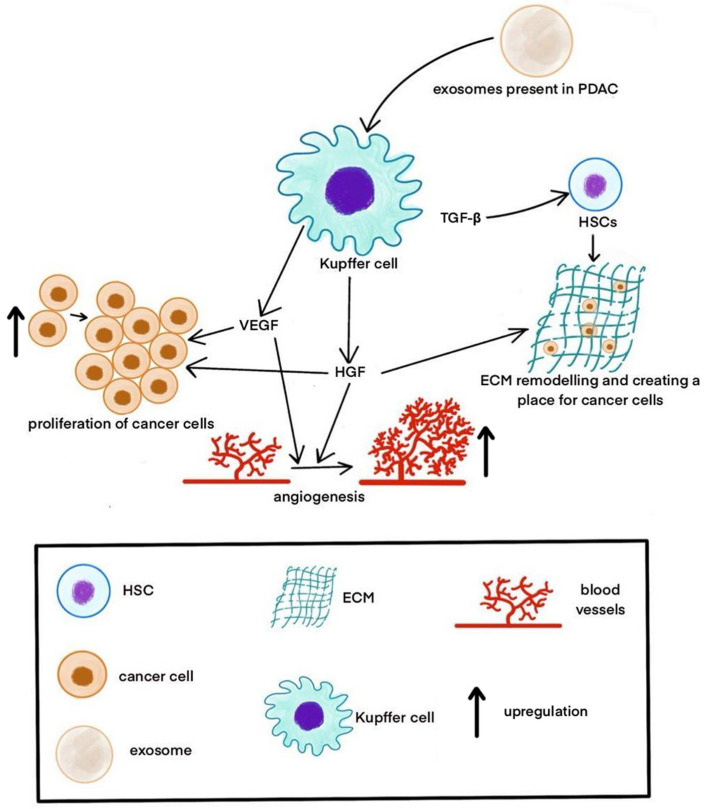
The role of Kupffer cells in metastasis in the liver. KCs may secrete VEGF and HGF. VEGF enhances the proliferation of cancer cells. HGF upregulates angiogenesis and ECM remodeling. TGF-β increases ECM remodeling through the activation of HSCs.

The ECM manages cancer cells’ proliferation, migration, adhesion, and differentiation [32]. ECM hosts many proteins, such as collagens, glycoproteins, proteoglycans, cytokines, and growth factors [33]. Regarding colorectal cancer (CRC) liver metastases, the hepatic ECM has a higher number of proteins, including type I, IV, and XII collagens, which participate in tumor proliferation [33,34,35]. A CRC model study showed that the citrullination of the ECM by peptidyl arginine deiminase 4 (PAD4) from CRC cells is a critical component of liver metastasis growth [33]. Type IV collagen, by managing CCL-7 and CCL5-5 chemokines, contributes to metastasis [32]. As for pancreatic ductal adenocarcinoma (PDAC) metastases to the liver, KCs play a pivotal role (Figure 1) [33]. KCs secrete hepatocyte growth factor (HGF) [35], which is an activated form that triggers the ECM remodeling, primarily through the fibronectin build-up, creating a place for PDAC cells [33]. KCs also secrete VEGF, mainly secreted in a fibrotic liver [36]. A PDAC model study demonstrated that exosomes present in PDAC express macrophage migration inhibitory factor (MIF). MIF then initiates TGFβ signaling in KCs, therefore causing an activation of HSCs and ECM remodeling [37]. TGFβ plays a vital role in fibrosis [38]. A niche suitable for liver metastasis is created by an influx of bone-marrow-derived macrophages caused by a collection of fibronectins [37]. HSCs release fibrillar collagen, proinflammatory cytokines, and profibrogenic cytokines, such as interleukin-1α, interleukin-1β, tumor necrosis factor (TNF-α), and prostaglandin E_2_ (PGE2) [36,39].

MET is a proto-oncogene that regulates cell growth, division, mobility, and differentiation [40]. The expression of MET in exosomes stimulates metastasis [39]. HGF moderates the mobility of epidermal cells [41]. HGF is proven to be a fibroblast-derived factor that plays a part in tumor migration [39]. HGF, as a ligand for the c-Met, binds MET [38] but does not activate it [39]. Metastasis is also stimulated by the epithelial–mesenchymal transition (EMT). Activated HGF/c-Met pathway causes EMT activation through a mitogen-activated protein kinase and phosphoinositide 3-kinase [42]. Furthermore, the HGF/c-Met pathway stimulates glucose transportation [43]. Additionally, neutrophil extracellular traps (NETs) induced by postoperative abdominal infectious complications lead to metastasis by the proliferation of gastric cancer cells. This mechanism is related to the EMT of gastric cancer cells. The EMT is also activated by TGFβ signaling [44]. EMT is characterized by the loss of E-cadherin, which can interact with c-Met [42].

## 4. Cell Adhesion

**Figure 2:**
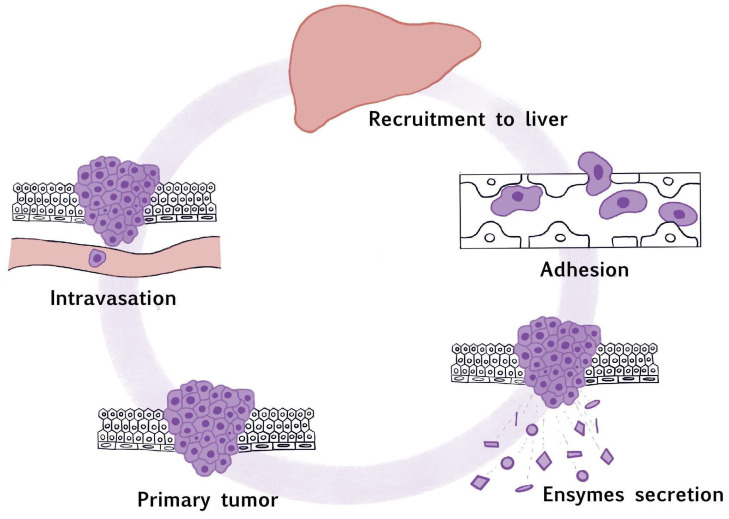
The role of the adhesion process in cancer cell migration [46]. Cancer cells from primary tumor recruit to the liver through intravasation. Afterwards, cancer cells undergo an adhesion process and then cancer cells secrete enzymes.

**Figure 3:**
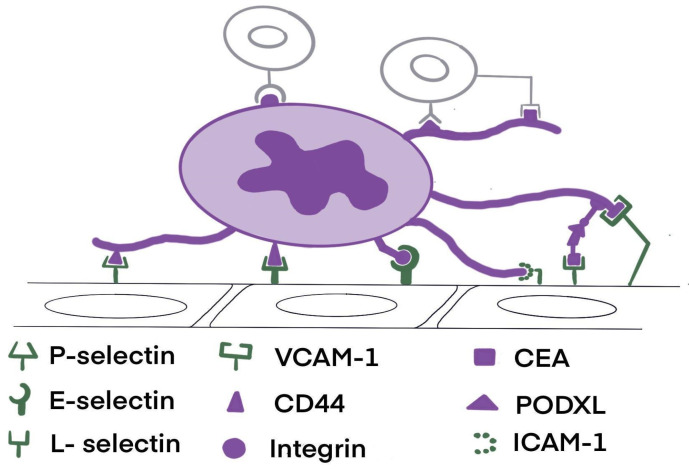
The molecules that play a role in cell adhesion mechanism [48]. Cancer cells may adhere through CD44, PODXL, and integrin interaction with VCAM-1 and the selectin family: P-selectin, E-selectin, and L-selectin.

Cell adhesion plays a crucial role in human physiology. It is essential for cell communication, regulation, differentiation, and migration. These processes also play a vital role in liver metastases (Figure 2). Cell adhesion can be divided into attachment and detachment. The molecules that play a role in this mechanism (Figure 3) are cell-surface proteins, which facilitate cell interactions or interactions between cells and the ECM. These proteins can be categorized into four main groups: cadherins, integrins, selectins, and members of the immunoglobulin superfamily [5]. Cell adhesion molecules (CAMs) play a significant role in liver metastasis.

Integrins facilitate interactions between cells and the ECM by binding to the ECM component proteins like fibronectin and other cells. This binding serves as a mediator for both cell–matrix and cell–cell interactions. Subsequently, this interaction triggers the activation of focal adhesion kinase (FAK), which, in turn, activates paxillin and talin. These proteins collectively regulate matrix adhesion and the spreading of tumor cells on the matrix [45].

Integrin αvβ6 plays a direct role in cellular movement by swiftly executing the endocytosis circulation process between the cell membrane and cytoplasm. Functioning as a significant receptor for tumor cells, integrin αvβ6 facilitates the cross-linking of the ECM and intracellular skeletal proteins, contributing to the advancement of tumors. This involvement encompasses tumor survival, adhesion to the basement membrane and ECM, and tissue penetration [45].

The selectin family comprises three members: P-selectin, E-selectin, and L-selectin. P-selectin is found in the storage granules of platelets (α-granules) and endothelial cells, allowing quick movement to cell surfaces when activated. On the other hand, L-selectin is consistently present on the cell surfaces of nearly all subpopulations of leukocytes [47]. E-selectin is a receptor for endothelial cell adhesion that is induced by cytokines. It plays a role in leukocyte rolling, ultimately leading to leukocyte transmigration. This mechanism is analogous to the transmigration observed in cancer cells [5].

P-selectin and E-selectin can attach to a group of molecules known as Sialyl-Lewis, found on specific tumor cells. The presence of this molecule on the cell surface is linked to metastatic potential and significantly influences liver metastasis [45].

ICAM-1 is regularly found in LSECs, hepatocytes, KCs, and HSCs within the liver. Its presence on endothelial cells is specifically involved in facilitating the adhesion of tumor cells to the endothelium. Furthermore, ICAM-1 can work with other adhesion molecules, such as VCAM-1, to enhance the adhesion of malignant cells [48,49,50].

E-cadherin hinders the onset of metastasis by promoting cell–cell cohesion in tumor cells. By fostering the aggregation of cells within the main mass, this molecule impedes the departure of tumor cells, consequently diminishing their metastatic capability. Decreasing E-cadherin and its related components allows tumor cells to detach from the primary site and initiate the metastatic process [45].

Cancer cells move through the vascular area by a sequence of less strong adhesive connections involving selectins found on endothelial cells and CD44, CEA, and PODXL on tumor cells’ surfaces. This rolling persists until the endothelium emits soluble adhesion molecules like VCAM1 and ICAM1. These molecules detain cells by engaging with integrin-facilitated cell–matrix adhesions, subsequently leading to the aggregation of neutrophils [51].

CD44 is excessively produced in various cell types, including cancer stem cells, and often exhibits alternative splicing variants believed to contribute to the development and progression of cancer. The primary binding partner for CD44 is hyaluronic acid (HA), a plentiful component of the ECM found in stromal and cancer cells. HA interacts with the ligand-binding domain of CD44, causing structural changes that facilitate the binding of adaptor proteins or cytoskeletal elements to intracellular domains. This, in turn, triggers multiple signaling pathways, resulting in cellular processes such as proliferation, adhesion, migration, and invasion. CD44v isoforms can also function as co-receptors by capturing and sequestering growth factors on the cell surface, subsequently presenting them to their specific receptors [52].

## 5. The Secretion of Proteolytic Enzymes

Liver metastasis is a complicated and multistep process. It can be divided into four major phases [52,53]: an intravasation, an extravascular proangiogenic, an angiogenic, and a growth phase [52]. Entry of CTCs into secondary or distant organ sites by mechanically entrapping in capillaries is a key step in metastasis [54,55]. Most CTCs become trapped in the capillary bed of a liver because of venous circulation from the intestine into the liver [54,56,57]. Afterward, CTSs are extravasated by breaching vascular walls or by forming an embolus, which can burst the vessel. It is worth noting that the blood vessel wall’s features may impact where cancer cells extravasate [54]. Those cells can extravasate into the organ parenchyma, where CTCs lead to the activation of proteases, promoting angiogenesis through the agency of MMPs. These processes often lead to micrometastases [56,57]. In this context, it is worth mentioning that extravasation depends on ECM remodeling, which emphasizes the role of MMPs [57]. Moreover, another essential stage in liver metastasis exists—the premetastatic niche, which can facilitate metastasis by outgrowing disseminated tumor cells [54,55,58,59].

**Figure 4:**
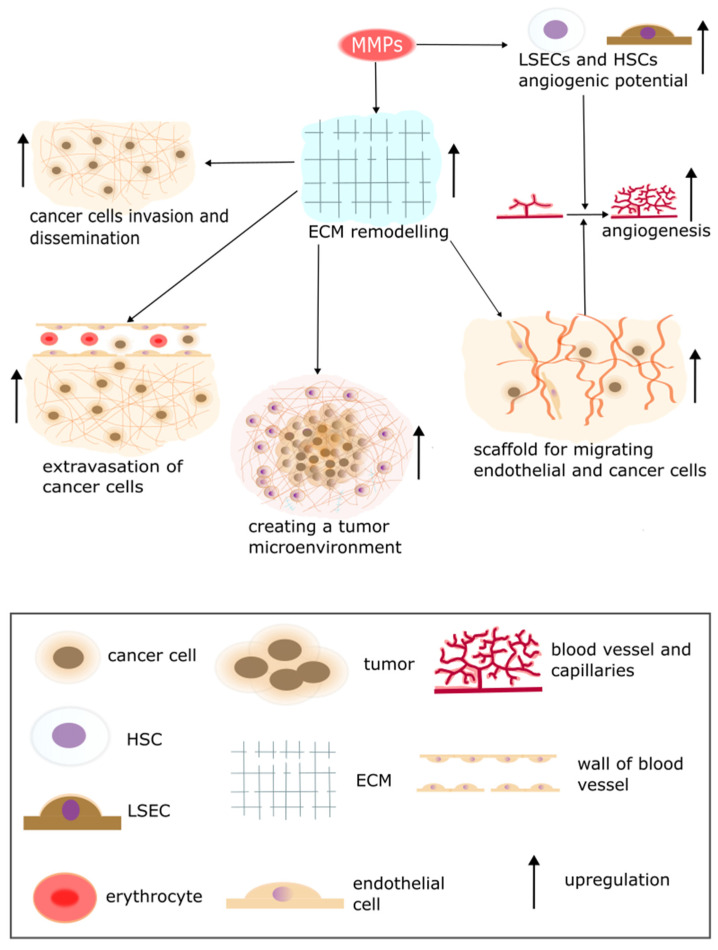
The role of MMPs in metastasis and tumorigenesis. ECM remodeling through MMPs secretion enhances cancer cells invasion, dissemination, and extravasation as well as creating a tumor microenvironment. MMPs create scaffolds for migrating endothelial and cancer cells through ECM remodeling. MMPs increase LSECs and HSCc angiogenic potential. The aforementioned processes lead to an increase in angiogenesis.

MMPs may contribute to creating a PMN, which is crucial in metastasis. Emerging evidence suggests that the premetastatic phase may facilitate liver colonization by spreading tumor cells [6]. Moreover, some tumors may create a PMN by remodeling the liver microenvironment to make it more hospitable to metastatic growth. It is essential because it occurs before malignant cells colonize this organ [60]. PMN may be formed by deposition of CTCs in the sinusoids and capillaries. Those cells secrete proteolytic enzymes, such as MMP-9, which may contribute to the formation of PMN [35]. Furthermore, MMPs may disrupt vasculature at the metastatic site, which may contribute to PMN development by induction of endothelial tip cells that produce TGF-β1 and ECM molecules. TGF-β leads to the transformation of fibroblasts into CAFs, which conduce to tumorigenic features of tumor microenvironment cells [61]. However, TGF-β may facilitate or impede tumorigenesis depending on the context [57]. TGF-β induces antitumorigenic M2 and N2 phenotype of tumor-associated macrophages (TAMs) and tumor-associated neutrophils (TANs) [62]. MMPs can also facilitate metastasis, for instance, by remodeling the ECM and ECM-binding receptor (Figure 4).

The role of proteolytic enzymes in metastasis in the liver is crucial because they influence the cytoskeleton. MMPs and cathepsins have meaningful functions during cytoskeletal rearrangements, which can facilitate cancer cell invasion and migration through stroma [54]. Moreover, MMPs degrade ECM and may release growth factors such as active forms of TGF-β through ECM degradation. MMPs can create a microenvironment which furthers tumor development through growth factors or cryptic binding site exposure [60]. In this context, it is worth mentioning that ECM is a crucial component of the tumor microenvironment [61,63]. MMPs are critical to maintaining ECM homeostasis [64,65] and significant in cancer cell invasion and dissemination [65]. Thus, MMPs might play a prominent role in metastasis to the liver [6,57] due to remodeling ECM [33,53,58,59,65]. Those enzymes play a significant role in metastasis by supplying a scaffold for migrating endothelial and cancer cells and encouraging angiogenesis [66,67]. It is worth mentioning that MMPs play a role in angiogenesis through promoting the angiogenic potential of liver cells, LSECs and HSCs [68]. Furthermore, MMPs may impact the results of treatment because high expression of MMPs is correlated with wrong prognosis and increases the risk of relapse [58,69,70]. Nonetheless, Peltonen et al. have shown better prognosis in patients with high expression of MMP-9 in colorectal tumor tissue and elevated preoperative myeloperoxidase. This study consisted of 111 patients with colorectal tumors and liver metastases. The serum concentration of MMP-9 and MPO was assessed using an enzyme-linked immunosorbent assay [71]. Moreover, tissue inhibitors of metalloproteinases (TIMP), which are specific inhibitors of MMPs, may impact ECM homeostasis. It seems paradoxical that a systemic increase in TIMP-1 renders the liver more liable to metastasis [70,72]. However, Cruz-Munoz et al. showed increased liver and kidney metastatic colonization by EL-4 lymphoma cells among timp-3-/- mice [73]. They play a role in liver cancer by bringing on the differentiation of liver fibroblasts into CAFs. Furthermore, TIMPs might impact creating a premetastatic niche in the liver by encouraging the homing of CTCs [55,59,69]. Thus, the role of TIMPs in metastasis in the liver should be better understood and further research is required. Another crucial role of MMPs is possibly activating dormant cancer cells. In an inflammatory environment, neutrophils may secrete neutrophil elastase and MMP-9, which leads to laminin degradation and subsequently results in exposure-specific laminin epitopes. Thus, activating an integrin-mediated cascade leads to the awakening of dormant cancer cells at sites of metastasis and, as a consequence, their proliferation [57].

It is worth mentioning that MMPs have a crucial function in colorectal cancer metastasis to the liver [53,56,57,65,69,74,75], which is significant because CRC is the most common cancer that metastasizes to the liver and one of the most common cancers in the world [52,53,76,77]. Colorectal cancer cells can secrete MMP-7, MMP-9, and MMP13, which positively correlate with liver metastases [57]. However, some studies show that only MMP9 was identified during colorectal liver metastasis (CRLM) [35]. This MMP contributes to the early expansion of CRC. It is important to note that the immune microenvironment plays a key role in CRLM [29,53]. TAMs have been divided into M1 and M2 phenotypes [53,78]. However, this dichotomy has historical importance. M2 TAMs secrete MMP9, which contributes to remodeling ECM, thus facilitating invasion and metastasis. It is also important to note that neutrophils, called TANs, play a role in CRC metastasis [53,56]. TANs can secrete MMP9, which facilitates angiogenesis and tumor invasion and supports cancer foci to expand [56,79]. It is worth mentioning that TAN and TAM are major sources of MMP9 in various murine models [56]. Also, CAFs of connective tissue cells can promote metastasis through the secretion of MMP2 and MMP9 [53,74]. Furthermore, CAFs can secrete MMP3, which can promote cancer cell invasion through the degradation of E-cadherin [66]. It is happening as a consequence of ECM remodeling [53]. Wernicke et al. have shown that MMP-13 expression was assessed in 137 biopsy samples from 105 patients with colorectal adenomas and CRC. MMP-13 expression was evaluated by Western blotting [69]. KCs can secrete MMP13. This is important because high expression of MMP13 is interlinked with a high rate of liver metastasis [65,69,75]. Furthermore, it also plays a role in poor prognosis and early relapse of disease [69]. However, it is worth noting that MMP23 is downregulated in liver metastasis in CRC [33]. Moreover, CRC metastasis to the liver is correlated with high systematic levels of TIMP1, which promotes the recruitment of neutrophils to the liver. These cells remodel the tissue, which leads to a tumor-permissive niche. Also, CTCs may cause an increase in TIMP-1 level [57].

It is worth noting that PDAC is often spread by metastasis to the liver [52,80]. The MMPs play a significant role in PDAC metastasis in the liver and outcome of this cancer. It was shown that the most common MMPs in PDAC are MMP1, MMP14, and MMP2, which are secreted by epithelial tumor cells, M2-like macrophages, and fibroblastic cells. The main target of MMPs is type I collagen (Col I). Hua et al. classified a cohort of 106 patients with resected PDAC. Survival data correlated with the tumor Col I state. Tumors enriched for matrix-metalloprotease-cleaved Col I (cCol I) have shown worse median survival in patients. Furthermore, high mRNA expression of MMPs is intercorrelated to the lesser survival of these patients [80]. Other important cells are stromal fibroblasts, which can increase the invasiveness of pancreatic cancer cells in mechanisms associated with elevated activation of MMP-2, facilitating cancer cell migration by degradation of ECM [70].

Non-small cell lung cancer (NSCLC) often metastasizes in the liver, which is significant because NSCLC accounts for >80% of lung cancer cases [70,81]. Several MMPs, such as MMP1, MMP2, and MMP9, have enhanced the risk of metastasis in lung cancer [82]. Moreover, during the preangiogenic phase, NSCLC metastasis in the liver, KCs, and neutrophils secrete MMPs and elastases. Furthermore, MMPs may conduct angiogenesis through several stages; ICAM-1 expressed on LSECs encourages the secretion of MMP2, which consequently induces HSCs to secrete VEGF-A and MMP2 [66,67]. It is worth mentioning that MMP2 has an important role in liver vascular homeostasis [66]. Those molecules facilitate metastasis by promoting the migratory and angiogenic potential of LSECs and HSCs. M2-type TAMs can also promote angiogenesis by secretion of MMP-2 and MMP-7 [67].

Uveal melanoma (UM) has a tendency towards metastasis in the liver [53,60,83], which is meaningful because, in most cases, the liver is the only stricken organ [60]. HSCs may be activated in the presence of metastatic cells. Consequently, they can promote desmoplastic stromal response and secrete MMPs and their inhibitors. These two processes lead to stiffness of the ECM around malignant lesions [60]. It is reflected in the treatment of this type of cancer because suppression of hepatic fibrosis might inhibit the development of hepatic metastasis [77]. Furthermore, there is a nodular growth pattern of metastasis in the liver. This pattern relies upon effaced or seldom infiltrated nodules of the tumor. These nodules can approximate or surround portal venules, which have an impact on surrounding hepatocytes by expression of MMP9 and VEGF. MMP can induce the proliferation of HSCs and allow melanoma cells to dissect through tissue planes. HSCs can develop into “pseudo-sinusoidal” spaces due to their proliferation. The above processes facilitate tumor nutrition, oxygenation, and nutrition but inhibit angiogenesis therein [83].

## 6. Methods for Inhibiting Liver Metastasis

### 6.1. Pharmacological Methods

Knowing the importance of the liver environment in metastasis progression, there are some pharmacological options that can prevent this process or at least delay it (Table 1). As was noted before, many cytokines promote the expansion of tumor cells. It is worth mentioning that anti-programmed cell death protein 1 (PD-L1) antibody therapy could be beneficial in some cases, especially if the administration is combined with other medication, especially VEGF inhibitors such as bevacizumab, as VEGF is an inductor of infiltration of tumor cells and angiogenesis [84,85]. Bevacizumab inhibits the binding of VEGF with its receptors and, therefore, it decreases VEGF stimulation, leading to limited blood supply to cancer cells [85]. Anti-VEGF therapy can also be expanded by providing other medications such as ramucirumab, regorafenib, and ziv-aflibercept later in the therapy. Firstly, the studies showed that the administration of ramucirumab or ziv-aflibercept in addition to chemotherapy consisting of infusional fluorouracil, leucovorin, and irinotecan (FOLFIRI) improved overall survival compared to patients who were administered FOLFIRI and placebo (13.3 versus 11.7 for ramucirumab and 13.5 versus 12.06 months for ziv-aflibercept). Secondly, regorafenib also improved the median overall survival in patients with metastatic CRC who progressed from standard therapy to the best supportive care compared to patients who received placebo (6.4 versus 5 months) [86,87,88,89]. The study showed that bevacizumab, as an addition to the therapy in patients with previously untreated metastatic CRC, prolongs the median survival duration. In this randomized study, 402 patients were administered irinotecan, fluorouracil, leucovorin (IFL), and bevacizumab, and the other 411 patients were administered IFL and placebo. The results showed that the median duration of survival was increased in patients who received IFL with bevacizumab compared to the group that received IFL and placebo (20.3 months to 15.6 months). What is more, the median duration of progression-free survival was also increased in the IFL plus bevacizumab group (10.6 months compared to 6.2 months in the IFL plus placebo group). In conclusion, adding bevacizumab to the therapy can improve both the median duration of survival and the median duration of progression-free survival [90]. It is worth mentioning that another study showed that antisense oligonucleotides (ASO) C-raf treatment attenuates E-selectin and reduces TNFα mRNA levels in mice, which are essential in metastases progression. The scientists showed that human colorectal carcinoma CX-1 cells under the influence of E-selectin can adhere to TNFα-activated murine hepatic sinusoidal endothelial cells [91,92]. To prove this, the mice were administered C-raf ASO and injected to receive CX-1 cells. As a result, the E-selectin expression was inhibited. It is worth mentioning that hepatic TNFα mRNA level was reduced only by 25%, leading the researchers to the conclusion that the inhibition of E-selectin is the main reason that contributes to reduced metastases formation in treated mice [92].

KCs should also be considered an additional target in therapy and metastasis prevention. Gadolinium chloride depletes KCs, resulting in a decreased number of liver tumor cells and VEGF-expressing infiltrating cells [93]. On the other hand, adjuvant immunotherapy, including the administration of tumor-specific monoclonal antibodies (Ab), can be beneficial as the therapy stimulates KCs, NK cells, and macrophages, resulting in potential cancer cell elimination [94].

The researchers discovered that administration of low doses of interferon-α (INF-α) promotes the stimulation of liver endothelial cells. Therefore, a barrier in blood vessels is formed, preventing tumor cell transmission to the liver and creating metastasis formation. However, the high doses of INF-α cause many side effects of the therapy and are less effective. Thereby, lower doses are recommended [95]. There is also an important cytokine called interleukin-12 (IL-12), which is crucial in antitumor immunity as it stimulates T helper cell 1 (Th1) and NK-cell differentiation. The systemic administration of IL-12 suggests that, in the tumor environment, there are cells that are responsive to IL-12, as the liver metastases were reduced after IL-12 administration, but there are still few reported clinical trials [96,97]. Due to its antiplatelet effect, lower doses of aspirin can also be used in antimetastatic therapy. As was mentioned before, the platelets play a crucial role in promoting metastasis as they protect the tumor cells and promote angiogenesis [15]. Aspirin is an inhibitor of cyclooxygenase-1 (COX1) and cyclooxygenase-2 (COX2) and, therefore, a negative regulator of prostaglandin E2, causing an inhibition of platelet aggregation. Moreover, prostacyclin biosynthesis is also inhibited after aspirin administration, which enhances the antiplatelet effect, as the prostacyclin controls the responses of platelets during pro-aggregatory stimulation [98,99].

Therapy consisting of herbs may also be considered during antimetastatic treatment. Natural antitumor products such as vinca alkaloids, vinblastine, and vincristine are often a choice in chemotherapy due to their antineoplastic effect [100,101]. Many studies focused on the pharmaceutical effects of herbal therapy and its antitumor role. The most promising herbs to prevent liver metastasis are curcumin, emodin, and honokiol because they present their anti-inflammatory effect. Firstly, they suppress IL-6 and nuclear factor-κB (NF-κB), known for their proinflammatory effect; secondly, they stimulate macrophages and dendritic cells; and, thirdly, they control the MMPs which take a role in tumor proliferation [22,102].

### 6.2. Other Liver Metastases Treatment Approaches

Pharmacological treatment of liver metastases is an effective way to deal with liver metastases. However, nonpharmacological treatments have significant applications. Hepatic metastasectomy is a crucial method to treat liver metastasis from UM. Mariani et al. have shown significantly improved long-term survival in selected patients using standard criteria for hepatic surgery. This study included 543 nonsurgical patients, of whom 255 had surgical intervention [103]. Surgery is offered to patients with liver metastases from UM at only 2% to 7% [104]. However, hepatic metastasectomy has a limited indication, local relapse is common, and survival is not increased compared with systemic therapy [105]. Moreover, surgical resection is the best treatment method for hepatic metastases from neuroendocrine tumors [106]. Alternative therapies, especially in patients not qualified for surgical treatment, include cryotherapy, radiofrequency ablation (RFA), and stereotactic radiotherapy [105]. RFA causes cellular death due to thermocoagulation necrosis but spares hepatic parenchyma [105,107]. RFA exhibits no difference in survival time and DFS regarding surgical resection among UM liver metastases patients [105]. RFA is a safety efficacy therapy. The major disadvantage is the high local recurrence rate [107]. RFA may not be performed if crucial structures in the liver, such as helium or large vessels, are near the tumor [108]. Thus, RFA may cause incomplete ablation of lesions near large vessels [107]. Another ablation method is microwave ablation (MWA) [107,109]. MWA exhibits a larger zone of necrosis than RFA. However, MWA may cause undesirable damage to structures near the tumor [107]. Hepatic transarterial embolization (TAE) consists of clogging the hepatic artery, which leads to ischemic and, as a consequence, the death of cancer cells. TAE is based on the fact that liver tumor superiorly receives blood from the hepatic artery. By contrast, normal tissue receives blood from a portal vein [106,109]. Bala et al. have analyzed that cryotherapy shows no difference in patients’ survival or recurrence of liver metastases compared to conventional liver metastases surgery [110].

## 7. Discussion

Our article aims to show that the liver metastatic cascade is a complex, multi-step biological process. The tumor microenvironment is pivotal in tumor progression and metastatic invasion [30]. To maximize the efficacy of novel targeted therapies, it is crucial to mitigate the protumorigenic functions and enhance the antitumorigenic functions of the tumor microenvironment. Understanding and predicting tumor progression, the development of distant metastases, and resistance to chemotherapy hinge on delineating the molecular mechanisms implicated in liver metastases. Further research is needed to identify factors within the tumor microenvironment that could be suitable targets for innovative therapeutic approaches [111].

The liver is a unique metabolic and immunological niche within the body. Its lymphatic system represents a complex anatomical organization with a large lymph output. Based on the repertoire of the biological functions associated with lymphatics and LECs (Figure 1), it is suggested that LEC expansion is not only a passive accompanying event during liver diseases. This is particularly intriguing because changes in LECs appear to reflect the type of peripheral inflammation—consequently, this area of research warrants further attention to elucidate its precise role in liver disease pathogenesis. Improved marker combinations enable the detection and sorting of these cells from the liver via flow cytometry. Combined with other techniques, such as histological analyses, this provides a robust foundation for further functional investigations. Advancing our understanding of liver diseases in this way could lead to novel therapeutic opportunities [9].

Metastasis emerges as the end-product of a multi-step cascade of events, including cancer cell invasion, intravasation, circulation, extravasation, and colonization into the target organs. It is responsible for the majority of cancer-related deaths worldwide. Recent studies indicate that communication among the immune system, the tissue, and cancer cells is vital in tumor and metastasis development. However, this communication and the mediators affecting the various metastatic steps remain unknown to a large extent [112].

Experienced surgeons and radiologists should evaluate patients with liver metastases from CRC that are potentially resectable because surgical resection remains the best treatment for long-term survival, although a minority of patients are amenable to the resection. Patients who are not suitable for surgical resection and who have no extrahepatic disease can be considered for regional therapies such as RFA and cryotherapy. Hepatic artery catheter chemotherapy and chemoembolization can be regarded as an alternative to systemic chemotherapy. Portal vein embolization may be combined in the treatment of patients with huge metastases. SIRT should be used for patients without extrahepatic metastases who failed in the treatment with 5-FU and other cytotoxic agents. Systemic chemotherapy should be administered in patients with extrahepatic diseases. Immunotherapy can only be used to amplify the efficacy of antitumor–cytotoxic agents in combination [4]. Treating liver metastases can include PDL-1 antibody therapy combined with bevacizumab [84,85]. As an adjuvant, bevacizumab can increase median survival time [102]. Combining FOLFIRI chemotherapy with ramucirumab, ziv-aflibercept, or regorafenib has been associated with a better prognosis [86,87,88,89]. The reduction in metastases formation can be managed by E-selectin inhibitors and low doses of INF-α [92,95]. The systemic administration of IL-12 as a treatment for liver metastases should be explored more since only a limited number of clinical trials have been reported [96,97].

Recent studies reveal the mechanisms that create a supportive environment for cancer cell colonization and growth in the liver. These studies highlight hepatocytes, HSCs, and KCs as key players in orchestrating myeloid cell accumulation and fibrosis. As a result, therapeutic strategies targeting specific molecular and cellular components of the liver’s prometastatic niche can potentially prevent liver metastasis and significantly improve patient outcomes [4]. The impact of cancer-derived MMPs on treatment should be a subject of further research as there are conflicting results on whether high MMP expression is correlated with poor prognosis [58,69,70,71].

Future studies should also prioritize understanding how the liver’s prometastatic niche affects adaptive immune responses against primary tumors and metastatic lesions. The observation that patients with liver metastases exhibit reduced responsiveness to immunotherapies suggests that the prometastatic niche in the liver may suppress T-cell responses both locally and systemically. This idea is further supported by the ability of SAA to regulate T-cell migration [4], which may impact T-cell infiltration into tumor tissue and subsequent interactions between T cells and tumor cells. Myeloid cells are also an important determinant of cancer dormancy. Therapeutic strategies targeting the liver’s myeloid cells may alter the recognition and elimination of tumor cells by T cells. Together, additional studies on mechanisms that direct the formation of a prometastatic niche and its impact on antitumor immune responses may lead to increased effectiveness of therapies for cancer [7].

## Figures and Tables

**Table 1:** Pharmacological methods used in liver metastases treatment.

Medication	Target of the Medication	The Pharmacological Effect
Bevacizumab	VEGF	Suppression of neovascularization
Ziv-aflibercept		Limited blood supply to cancer cells
Regorafenib	VEGFR	Suppression of neovascularization
ramucirumab		Limited blood supply to cancer cells
Antisense oligonucleotides (ASO) C-raf treatment	E-selectin,	Reduction in metastases formation
	TNFα mRNA	
Gadolinium chloride	KCs	Decreased number of VEGF-expressing infiltrating cells
Tumor-specific monoclonal antibodies (Ab)		Potential cancer cell elimination
INF-α	Liver endothelial cells	Reduction in metastases formation
IL-12	T helper cell 1 (Th1) NK-cells	Antitumor immunity stimulation
Aspirin	COX1	Inhibition of platelet aggregation
	COX2	
Vinblastine	Tumor cells	Antineoplastic effect
Vincristine		
Curcumin	IL-6	Proinflammatory effect
Emodin	Nuclear factor-κB (NF-κB)	Suppression of tumor stimulation
Honokiol	Macrophages	Antitumor immunity stimulation
	Dendritic cells	
	MMPs	
